# The influence of conceptual concreteness on the reading acquisition and integration of novel words into semantic memory via thematic relations

**DOI:** 10.3389/fpsyg.2023.1132039

**Published:** 2023-05-11

**Authors:** Jinfeng Ding, Panpan Liang, Xinyu Guo, Yufang Yang

**Affiliations:** ^1^CAS Key Laboratory of Behavioral Science, Institute of Psychology, Beijing, China; ^2^Department of Psychology, University of Chinese Academy of Sciences, Beijing, China

**Keywords:** concreteness, thematic relation, novel word learning, contextual reading, EEG

## Abstract

Plenty of studies have been conducted to reveal neurocognitive underpinnings of conceptual representation. Compared with that of concrete concepts, the neurocognitive correlates of abstract concepts remain elusive. The current study aimed to investigate the influence of conceptual concreteness on the reading acquisition and integration of novel words into semantic memory. We constructed two-sentence contexts in which two-character pseudowords were embedded as novel words. Participants read the contexts to infer the meaning of novel words which were either concrete or abstract, and then performed a lexical decision task and a cued-recall memory task. In lexical decision task, primed by the learned novel words, their corresponding concepts, thematically related or unrelated words as well as unlearned pseudowords were judged whether they were words or not. In memory task, participants were presented with the novel words and asked to write down their meaning. The contextual reading and memory test can demonstrate the modulation of conceptual concreteness on novel word learning and the lexical decision task can reveal whether concrete and abstract novel words are integrated into semantic memory similarly or not. During contextual reading, abstract novel words presented for the first time elicited a larger N400 than concrete ones. In memory task, the meaning of concrete novel words was recollected better than abstract novel words. These results indicate that abstract novel words are more difficult to acquire during contextual reading, and to retain afterwards. For lexical decision task behavioral and ERPs were graded, with the longest reaction time, the lowest accuracy and the largest N400s for the unrelated words, then the thematically related words and finally the corresponding concepts of the novel words, regardless of conceptual concreteness. The results suggest that both concrete and abstract novel words can be integrated into semantic memory via thematic relations. These findings are discussed in terms of differential representational framework which posits that concrete words connect with each other via semantic similarities, and abstract ones via thematic relations.

## 1. Introduction

Concepts are a component of knowledge system and play an important role in human cognition. It is well-established that concrete concepts are mainly represented by sensory-motor information (for reviews see Capitani et al., 2003; Binder and Desai, 2011; Lambon Ralph et al., 2017). But the representation of abstract concepts – concepts that have no perceivable referents – remains elusive (but see Jessen et al., 2000; Binder et al., 2005; Wang et al., 2010; Pulvermüller, 2013; Hoffman et al., 2015; Binder, 2016; Borghi, 2022). One of the debated topics in this area is whether the manner of connections with other concepts is different for concrete and abstract concepts. The current study addressed the issue by examining how concrete and abstract novel words are learnt in contextual reading and integrated into semantic memory. To this end, we simulated vocabulary learning in second language (L2) study by associating novel words with known concepts in contexts, a crucial way to novel word learning (Batterink and Neville, 2011; Elgort and Warren, 2014). The findings will shed new light on the influence of conceptual concreteness on L2 reading acquisition.

### 1.1. Concreteness effect in word acquisition

Previous studies have shown that concrete words are learned better over abstract words in first language (L1, e.g., Palmer et al., 2013) and L2 (e.g., De Groot and Keijzer, 2000; Elgort and Warren, 2014) acquisition. For instances, Palmer et al. (2013) explored the influence of conceptual concreteness on the acquisition and subsequent processing of novel words. They asked native English speakers to learn real but rare English words which were paired with their definitions. It was found that participants acquired concrete words better than abstract words as indicated by the higher accuracy in the word-definition pairing test for the former ones. Meanwhile, participants responded faster to concrete words compared to abstract words in the later semantic categorization and lexical decision tasks, indicating a processing advantage of concrete (vs. abstract) words.

In L2 vocabulary learning, using a paired-associate learning in which English pseudowords were paired with Dutch translations, De Groot and Keijzer (2000) found that concrete novel words were easier to learn and less susceptible to forgetting for native speakers of Dutch, as indicated by higher recall accuracies for concrete words compared to abstract words. In contextual reading, Mestres-Missé et al. (2014) examined the influence of conceptual concreteness on novel word learning. They asked participants to read sentences ending with novel words and found that the reading times in the second (vs. first) sentences increased for abstract novel words but decreased for concrete novel words. These results indicated that the meaning of concrete words was discovered and learned faster than abstract words.

The concreteness advantage effect in word learning could be accounted for by dual coding theory in which an imagery-based system and a verbal-based system are associated with concepts in semantic memory (Paivio, 1986, 1991). According to the dual coding theory, while both the imagery-based and verbal-based systems are associated with concrete words, only the verbal-based system is involved for abstract words. With the support of the two systems, concrete words were learned better than abstract words. The neuroanatomical structure for dual-coding knowledge has been depicted by the neural framework of Bi (2021), in line with the direct neural evidence provided by previous study (Mestres-Missé et al., 2009). In the study, participants were asked to read sentences and to derive the meaning of novel words associated to concrete and abstract concepts. The important finding was that the ventral anterior fusiform gyrus, a region associated with high-level visual processing, showed a selective activation for novel concrete words, indicating the involvement of the imagery-based/sensory-derived system in concrete word learning.

Besides the dual coding theory, the concreteness effect could also be explained by context availability hypothesis (Schwanenflugel and Shoben, 1983; Schwanenflugel and Stowe, 1989). This theory argues that the difference between concrete and abstract words could be attributed to the discrepancy in the availability of contextual information. That is, abstract words tend to appear in a wider range of contexts than concrete words, which results in a looser contextual constraint for the abstract words (Hill et al., 2014). Based on this theory, the context wherein concrete and abstract concepts appear will modulate the concreteness effect. For instance, the concreteness effect in lexical decision task was absent after concrete and abstract words were processed in sentences (Schwanenflugel et al., 1988; van Hell and de Groot, 2008), showing a facilitation effect of sentence context on the processing of abstract words. The stronger contextual facilitation to abstract words processing than concrete words was also observed in other studies (Hoffman et al., 2015; Bechtold et al., 2021, 2022). However, in contrast to this theory, Taylor et al. (2019) found that concreteness effects were reliable for words presented both in isolation and in contexts, and similar for words in low-and high-constraint contexts.

Therefore, how the conceptual concreteness influences word acquisition in sentence reading needs to be further explored. If the facilitation effect stems from the context availability, there should be no difference between the acquisition of concrete and abstract novel words in sentence reading. In contrast, according to the dual coding theory the superiority of concrete concepts in learning through sentence reading should be observed.

### 1.2. Semantic integration of novel words

Previous studies have shown that, in contextual reading, novel words can be rapidly learned and integrated into the semantic network via different semantic relations (Borovsky et al., 2012; Chen et al., 2014; Ding et al., 2017a; Zhang et al., 2017, 2019). Among these studies, two important semantic relations, taxonomic and thematic relations (Mirman et al., 2017), are mainly explored. Taxonomic relation is established on the bases of semantic similarities between concepts (Murphy, 2010), and thematic relation organizes concepts by their complementary roles in the same event or episode (Estes et al., 2011).

In contextual reading, the experiments on novel word learning usually include a learning phase and a following testing phase. In the learning phase, novel words are embedded in contexts and participants are asked to read the contexts for deriving the meaning of novel words incidentally or intentionally. In the testing phase, the novel words serving as primes are paired with different types of semantically (taxonomically or thematically) related or unrelated target words. Participants are asked to judge whether the target word is a word or not. By comparing the behavioral and neural responses to the different types of target words, researchers could examine whether the novel words have been integrated into semantic memory through certain semantic relations.

Using the contextual learning paradigm, it is found that when contexts provide semantic features of concepts, the taxonomically but not thematically related words could be primed by the novel words, as indicated by the smaller N400s for the taxonomically related words than the unrelated words (Ding et al., 2017a). The results suggest that semantic integration of novel words into semantic memory is through taxonomic relations but not thematic relations (Ding et al., 2017a). However, when contexts describing events or scenarios were provided in the learning phase, the novel words could prime both taxonomically and thematically related words, indicating semantic integration of novel words into semantic memory via both taxonomic and thematic relations (Zhang et al., 2017, 2019). Taken together, the results suggest that the information provided by learning contexts influences the way of novel words integration into semantic memory.

Most of the aforementioned contextual reading studies are concerned with concrete concepts. How novel words associated with abstract concepts are integrated into semantic memory needs to be addressed. An influential theory, different representational frameworks (Crutch and Warrington, 2005, 2007; Crutch et al., 2006), assumes that concrete concepts are organized by semantic similarity and abstract ones by semantic association. In other words, concrete concepts connect with other concepts via taxonomic relations, and abstract ones via thematic relations. The theory stemmed from the results of a series of neuropsychological studies with patients. In the first study (Crutch and Warrington, 2005) a patient with semantic refractory assess deficit was asked to perform a task of matching spoken word to a written one. The target written word was paired with other three taxonomically (similarity-based) or thematically (association-based) related words. The results revealed a taxonomic but not thematic interference for concrete words, and a reverse pattern for abstract ones. The different representations for concrete and abstract concepts are also confirmed by studies using different tasks or techniques in normal individuals (Crutch et al., 2009; Duñabeitia et al., 2009). However, the findings were not replicated by some subsequent studies (e.g., Hamilton and Coslett, 2008; Zhang et al., 2013; Geng and Schnur, 2015). For instance, Geng and Schnur (2015) also used a spoken word to written word matching task and reported higher error rates and longer reaction times for both taxonomically and thematically related words compared with unrelated words irrespective of the concreteness of the target words. In addition, the interference was larger for the taxonomic than thematic words, indicating the prominent role of taxonomic relation in the organizations of both concrete and abstract concepts (Xu et al., 2018).

Given the abovementioned debate, how abstract novel words are integrated into semantic memory after contextual reading needs to be further explored. Ding et al. (2017b) examined whether concrete and abstract novel words learnt through contextual reading could be integrated into semantic memory via taxonomic relations by using event-related potential (ERP) technique with paradigm similar to previous studies (e.g., Chen et al., 2014; Ding et al., 2017a; Zhang et al., 2017, 2019). Participants were asked to derive the meaning of novel words through reading episodic contexts and performed a lexical decision task with novel words as semantic primes. The results showed that both concrete and abstract novel words could prime their taxonomically related words, indicating that both concrete and abstract novel words can be integrated into semantic memory via taxonomic relations, which was compatible with Geng and Schnur (2015).

However, the taxonomic and thematic relations are not mutually exclusive (Hill et al., 2014), that is, the taxonomically related words could also be thematically related with each other. Moreover, thematic relations are more dominant to abstract words (Zhang et al., 2013; Troyer and McRae, 2022). For example, Zhang et al. (2013) found both categorical (taxonomic) and associative (thematic) blocking interference for abstract words, but only categorical blocking interference for concrete words, indicating more relevance of abstract concepts to thematic relations. It is, hence, needed to further examine the (a)symmetry of semantic integration of concrete and abstract novel words via taxonomic and thematic relations.

Based on the aforementioned studies, the current study aimed to explore how the concreteness of concepts influences word acquisition during contextual reading, and whether the concrete and abstract novel words could be integrated into semantic memory via semantic relations in a similar way.

## 2. Research methodology

### 2.1. Research questions and theoretical hypotheses

The study aimed to address two research questions. Whether the concrete novel words could be learned better than the abstract ones in contextual reading? After contextual reading, whether the concrete and abstract novel words could be integrated into semantic memory through thematic relations in the same way? The two questions were examined through three experimental tasks. First, in the context reading phase, EEG data were recorded when participants read contexts to explore the difference in brain responses to concrete and abstract novel words in thematic contexts. Second, in the testing phase, lexical decision task was used to test how the concrete and abstract novel words were integrated into semantic memory when primed by the novel words. Third, a cued-recall memory test was conducted to explore the effect of concreteness on memory consolidation. The cued-recall memory test being conducted after the lexical decision task is to exclude the influence of explicit semantic retrieval of novel words on the implicit semantic priming to their corresponding concepts. In sum, the results of contextual reading and cued-recall memory tasks would show the learning effects for novel words, and the lexical decision task further elucidate integration processes of novel words into semantic memory.

As to the effect of the conceptual concreteness on word acquisition during contextual reading, in terms of the dual coding theory (Paivio, 1986, 1991), the concrete novel words would be learned better than the abstract novel words with the support of both verbal-and imagery-based systems, which would be reflected by smaller N400s and/or higher cued-recall accuracy for the concrete (vs. abstract) novel words. However, on the basis of the context availability hypothesis (Schwanenflugel and Shoben, 1983; Schwanenflugel and Stowe, 1989), the learning effects would be of no difference for the concrete and abstract novel words since the learning contexts provided equal context availability for them.

For the integration of concrete and abstract novel words into semantic memory via thematic relations, it was predicted that the novel words would connect with the thematically related words as indicated by the behavioral and/or neural semantic priming effects, namely, smaller N400s, shorter reaction time, and/or higher accuracy for the thematically related targets than for the unrelated targets, based on previous studies (e.g., Zhang et al., 2017, 2019). If the different representational framework (Crutch and Warrington, 2010) applies to the learning process, the semantic priming effects would be different in the two conditions. In contrast, if concrete and abstract concepts connect with other concepts through similar semantic relations, similar semantic priming effects in the concrete and abstract conditions would be observed.

### 2.2. Participants

Twenty-four university students (mean age 21.4 years, range 18–27, 12 males), right-handed native speakers of Chinese, took part in the experiment. They did not report dyslexia or neural impairment with normal or corrected-to-normal vision. This research was approved by the Ethics Committee of Institute of Psychology, Chinese Academy of Sciences. Before the EEG experiment, all subjects were given a written informed consent in accordance with the principles of the Declaration of Helsinki.

### 2.3. Materials

The materials in the learning phase were the same with those used in our previous study (Ding et al., 2017b). Sixty-six two-character pseudowords which can be pronounced but lack meaning served as novel words. They were embedded in two-sentence contexts to represent known concepts, half of which are concrete and the other half abstract. The novel words in the first sentences were always at the sentence-final position (see Table 1 for an example of stimuli). According to the pretest results (Table 2), most of participants could infer the meaning of the novel words in the two conditions with no difference between them [*t*_(64)_ = 0.20, *p* = 0.840]. In addition, most of participants expected the corresponding concepts of the novel words at the terminal positions of the first sentences with equally high cloze probabilities [*t*_(64)_ = −0.10, *p* = 0.924]. The results showed that the sentence constraints were not significantly different for concrete and abstract conditions, therefore excluding the potential influence of sentence constraint on the concreteness effect (Taylor et al., 2019; Bechtold et al., 2022). In addition, considering the potential influence of sentence complexity on word recognition, we used sentences with simple structures and both the numbers of characters [Concrete: *Mean* (*SD*) = 33.67 (2.34); Abstract: *Mean* (*SD*) = 33.64 (2.66), *t*_(64)_ = 0.05, *p* = 0.961] and words [Concrete: *Mean* (*SD*) = 12.00 (1.09); Abstract: *Mean* (*SD*) = 11.94 (1.20), *t*_(64)_ = 0.22, *p* = 0.830] were matched between the concrete and abstract conditions.

The lexical decision task was performed in the semantic priming paradigm, with the novel words serving as primes. Three types of real words: the corresponding concepts of novel words (CC), thematically related words (TR), and unrelated words (UR) and three pseudowords served as target words. Sixteen participants (mean age 22.7 years, range 19-27, 8 males) rated the semantic relatedness of the TR and UR targets to the CC targets on a 7-point Likert scale (7 indicates the most closely related and 1 indicates unrelated). Since the novel words were pseudowords, their concreteness was assigned based on that of their corresponding concepts. Sixteen participants (mean age 22.5 years, range 19-27, 8 males) rated the concreteness of all the target words on a 7-point Likert scale (7 indicates the most concrete, 1 indicates the most abstract). In addition, 21 participants (mean age 22.5 years, range 18-29, 11 males) rated the valence (7 indicates the most positive, 1 indicates the most negative) and arousal (7 indicates the highest arousal, 1 indicates the lowest arousal) of all the target words on 7-point Likert scales. Meanwhile, word frequency (Cai and Brysbaert, 2010) and number of strokes of all the target words were calculated. The descriptive results of all the aforementioned pretests are presented in Table 2. As can be seen in Table 3, the statistical analyses results indicated that the TR targets were more semantically related to the CC targets than the UR targets irrespective of the conceptual concreteness. In addition, the concrete words are rated to be more concrete than the abstract words irrespective of the target condition. Besides, all the target words were not significantly different in valence, arousal, word frequency, or number of strokes.

Meanwhile, for the TR targets, we calculated their cloze probabilities in the first sentences and their inferring probabilities in the whole discourses. Due to the non-normal distribution of the ratios, we conducted Mann-Whitney tests and found that the cloze probabilities [*n* = 66, *z* = -1.00, *p* = 0.317] or the inferring probabilities [*n* = 66, *z* = -1.00, *p* = 0.317] of the TR targets were not significantly different between the concrete and abstract conditions, indicating that participants learned the correct meaning of novel words.

**Table 1:** Examples of the stimuli in the learning phase and lexical decision task.

Learning discourses in the learning phase		
Concrete condition	Abstract condition	
小蝌蚪长大之后会变成一只**芋沌**，此刻池塘里的荷叶上蹲着一只**芋沌**在捕食昆虫。 A little tadpole grows up into a **yudun**. Right now on a lotus leaf there is a **yudun** catching insects.	获得诺贝尔奖是科学家的最高**栗芸**，大家都在为赢得这份最高的**栗芸**而努力。 To a scientist wining the Nobel prize is the highest **liyun**. Everyone is trying hard to win this highest **liyun**.	
Targets in the lexical decision task		
	Concrete condition	Abstract condition
Corresponding concept (CC)	**青蛙**(frog)	**荣誉**(honor)
Thematically related word (TR)	稻田(paddy)	掌声(applause)
Unrelated word (UR)	裤子(pants)	说法(statement)
Pseudoword	晾岌(liang ji)	贡颠(gong dian)
Pseudoword	甚筋(shen jin)	募旺(mu wang)
Pseudoword	泉愧(quan kui)	屑泊(xie bo)

The original stimuli are Chinese followed by English translations. The novel words are in boldface in the discourses.

**Table 2:** Means (*SD*s) of the stimuli properties.

	Corresponding concept		Thematically related word		Unrelated word	
	Abstract	Concrete	Abstract	Concrete	Abstract	Concrete
Relatedness	–	–	5.97 (0.47)	5.95 (0.63)	2.03 (0.38)	1.86 (0.50)
Concreteness	2.73(0.56)	6.15 (0.55)	2.74 (0.64)	5.95 (0.61)	2.53 (0.42)	6.03 (0.55)
Valence	4.55 (0.95)	4.57 (0.81)	4.62 (0.83)	4.45 (1.00)	4.24 (0.54)	4.55 (0.71)
Arousal	3.61 (0.87)	3.43(0.80)	3.50 (0.96)	3.43 (1.00)	3.37 (0.96)	3.45 (1.18)
Word frequency	2.52 (0.89)	2.57 (0.97)	2.62 (0.84)	2.26 (0.75)	2.34 (0.86)	2.51 (0.73)
Number of strokes	16.72 (5.10)	15.61 (4.77)	16.89 (5.19)	17.06 (4.87)	16.09 (4.08)	17.00 (3.62)
Cloze probability	81.06% (20.86%)	80.80% (21.60%)	0	0.25% (1.45%)		
Inferring probability	96.72% (5.49%)	96.97% (4.57%)	0	1.52% (8.70%)		

Cloze probability represents the proportion of the participants who gave the target words to complete the first sentences which were presented without the last words. Inferring probability represents the proportion of the participants who gave the target words as the meaning of novel words. SD: standard deviation.

**Table 3:** *F*-values of the ANOVAs on the stimuli properties.

	Relatedness	Concreteness	Valence	Arousal	Frequency	Strokes
Target condition	1662.07***	1.46	0.85	0.23	1.03	0.50
Conceptual concreteness	1.52	1502.16***	0.17	0.16	0.02	< 0.001
Target condition by Conceptual concreteness	0.51	1.32	1.72	0.31	2.24	0.83

The dfs of target condition and the interaction between target condition and conceptual concreteness are (1, 64) for relatedness, and (2, 128) for other properties. The df of conceptual concreteness is (1, 64) for all the properties. *** Significant at.001 level.

### 2.4. Procedure

Participants who were not recruited in any pretests took part in EEG experiment. The 66 discourses were divided into six blocks with each of three blocks including 10 discourses (five concrete and five abstract) and each of the other three blocks including 12 discourses (six concrete and six abstract). In each block, participants read the contexts in a pseudo-random order with no more than three discourses of the same condition being presented consecutively. Following a 1,000-ms fixation cross in the center of the screen, the contexts were presented one word or two-word phrase at a time (500-ms duration, 300-ms inter-stimulus interval (ISI)). The novel words were always presented in isolation for 1,000 ms. The two-sentence discourse was presented again on the screen after the last phrase and participants could press the space button if they had inferred the meaning of the novel words. After a 2000-ms resting screen, the next trial began.

After reading all 10 or 12 discourses in a block, participants performed a lexical decision task. Following a 1,000-ms fixation cross in the center of the screen, a prime word and a target word were presented in serial for 300 ms with a 200-ms ISI. Participants pressed the “F” or “J” on the keyboard to indicate whether the target word was a real word or not as quickly and accurately as possible. The button press was counterbalanced across participants. The word pairs were presented in a pseudo-random order: the same trial type was not presented consecutively more than three times and word pairs containing the same novel word were not presented in succession.

There was a short break between blocks. Approximately 20 min after all the blocks, participants performed a cued-recall memory task in which they were asked to write down the meaning of novel words learned in the experiment. Before the EEG experiment, participants had been told that there would be a memory test for the novel words.

### 2.5. Electrophysiological recording and Preprocessing

EEG was recorded (0.05–100 Hz, sampling rate 500 Hz) during contextual reading and lexical decision task with 64 Ag/AgCl electrodes, with the online reference linked to the right mastoid electrode. Vertical blinks and horizontal eye-movements were monitored. All electrodes impendence was kept below 5 kΩ. With NeuroScan software 4.5, automatic correction of the ocular artifacts (Semlitsch et al., 1986) and a band-pass filter at 0.1–30 Hz were conducted. Then the EEG data were segmented into 1,200-ms epochs from 200 ms before to 1,000 ms after the onset of critical words. After baseline correction with the mean amplitudes of prestimulus interval, an artifact correction was performed with criteria of ±80 μV at all electrodes except for electrooculograms, and the ERPs were re-referenced offline to the algebraic average of two mastoids.

### 2.6. ERP data analysis

Based on previous studies (Ding et al., 2017b) and visual inspections, 300–400 ms (for learning phase)/300–500 ms (for testing phase) and 500–1,000 ms (for both learning and testing phases) time windows were selected for the N400 and late components, respectively. For the learning phase, conceptual concreteness (abstract, concrete), presentation time (first, second), laterality (left, middle, right), and anteriority (anterior, central, posterior) served as within-subject factors in repeated measures ANOVAs. For the testing phase, conceptual concreteness (abstract, concrete), target condition (CC, TR, UR), laterality and anteriority were taken as within-subject factors in repeated measures ANOVAs. The combination of laterality and anteriority resulted in nine regions of interest with each containing six electrodes: left anterior (F3, F5, F7, FC3, FC5, and FT7), left central (C3, C5, T7, CP3, CP5, and TP7), left posterior (P3, P5, P7, PO5, PO7, and O1), middle anterior (F1, FZ, F2, FC1, FCZ, and FC2), middle central (C1, CZ, C2, CP1, CPZ, and CP2), middle posterior (P1, PZ, P2, PO3, POZ, and PO4), right anterior (F4, F6, F8, FC4, FC6, and FT8), right central (C4, C6, T8, CP4, CP6, and TP8), and right posterior (P4, P6, P8, PO6, PO8, and O2). In addition, for the pair-wise comparisons involving factors with three levels, corrected *p* values were reported with Bonferroni correction.

## 3. Results

### 3.1. Behavioral data

Due to the non-normal distribution of the data, generalized linear mixed-effects model analyses were conducted in R (R Core Team, 2013) using glmer function for the reaction time (RT) with a logarithmic link function (Lo and Andrews, 2015) and accuracy (ACC). Meanwhile, hypr package (Rabe et al., 2020) was used to construct custom contrast coding mainly for the pair-wise comparisons for the target condition. In the models, conceptual concreteness, target condition and their interaction were included as fixed effect factors. The models were initially constructed with maximal random effect structures. When the models failed to converge, we simplified the random structure according to Bates et al. (2015). The final models for the RT and ACC were the same:


$$\mathrm{RT}/\mathrm{ACC}\sim {\mathrm{concreteness}}^{*}\mathrm{condition}+\left(1\mathrm{|subject}\right).$$

**Figure 1:**
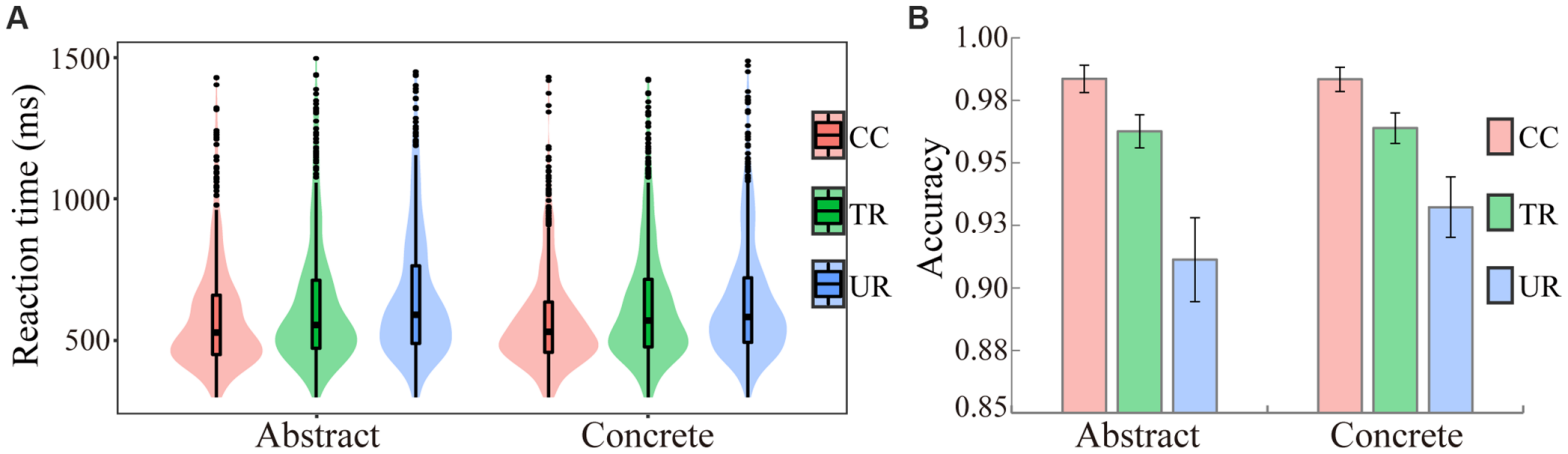
The reaction times of the correct responses **(A)** and the accuracies **(B)** for target words in each condition. CC: corresponding concepts; TR: thematically related words; UR: unrelated words.

For the reaction time, error trials (4.31%) and outlier data points (beyond 2.5 *SD*s as well as shorter than 300 ms or longer than 1,500 ms, 4.88%) were excluded from the analysis. Figure 1A presents the reaction time of the remained trials in each condition. Table 4 presents the results of custom contrast coding for the reaction time. The results showed that participants responded fastest to the CC targets, then to the TR targets and slowest to the UR targets. Neither the main effect of conceptual concreteness nor the interaction between conceptual concreteness and target condition were significant.

**Table 4:** GLMM results for the reaction time.

	Estimate	Std. Eror	*t* value	Pr (>|t|)
(Intercept)	−1.78	0.120	−14.78	< 2e-16 ***
Conceptual concreteness	0.012	0.011	1.16	0.245
CC vs. UR	−0.182	0.013	−13.95	< 2e-16 ***
TR vs. UR	−0.056	0.012	−4.39	1.16e-05 ***
CC vs. TR	−0.126	0.013	−9.69	< 2e-16 ***
Abstract (CC-UR) vs. Concrete (CC-UR)	−0.002	0.013	−0.22	0.830
Abstract (TR-UR) vs. Concrete (TR-UR)	−0.022	0.013	−1.68	0.823
Abstract (CC-TR) vs. Concrete (CC-TR)	0.019	0.013	1.45	0.147

CC: corresponding concepts; TR: thematically related words; UR: unrelated words.

Table 5 presents the results of custom contrast coding for the accuracy. As also can be seen from Figure 1B, the accuracy decreased in a graded manner from the CC targets, to the TR targets, finally to the UR targets. Similar to the results for the reaction time, the main effect of conceptual concreteness or the interaction between conceptual concreteness and target condition were not significant.

**Table 5:** GLMM results for the accuracy.

	Estimate	Std. Eror	z value	Pr (>|z|)
(Intercept)	3.48	0.17	19.97	< 2e-16 ***
Conceptual concreteness	−0.11	0.17	−0.65	0.516
CC vs. UR	1.63	0.22	7.49	7.13e-14 ***
TR vs. UR	0.81	0.16	4.95	7.28e-07 ***
CC vs. TR	0.81	0.24	3.43	0.000598 ***
Abstract (CC-UR) vs. Concrete (CC-UR)	0.15	0.22	0.68	0.498
Abstract (TR-UR) vs. Concrete (TR-UR)	0.13	0.16	0.78	0.43
Abstract (CC-TR) vs. Concrete (CC-TR)	0.02	0.24	0.08	0.937866

CC: corresponding concepts; TR: thematically related words; UR: unrelated words.

In the cued-recall memory test, participants recollected the meaning of concrete novel words better than that of abstract novel words [Concrete: *Mean* (*SD*) = 12.29 (10.93); Abstract: *Mean* (*SD*) = 9.38 (9.88), *t*_(23)_ = 4.34, *p* < 0.001].

### 3.2. ERP data

**Figure 2:**
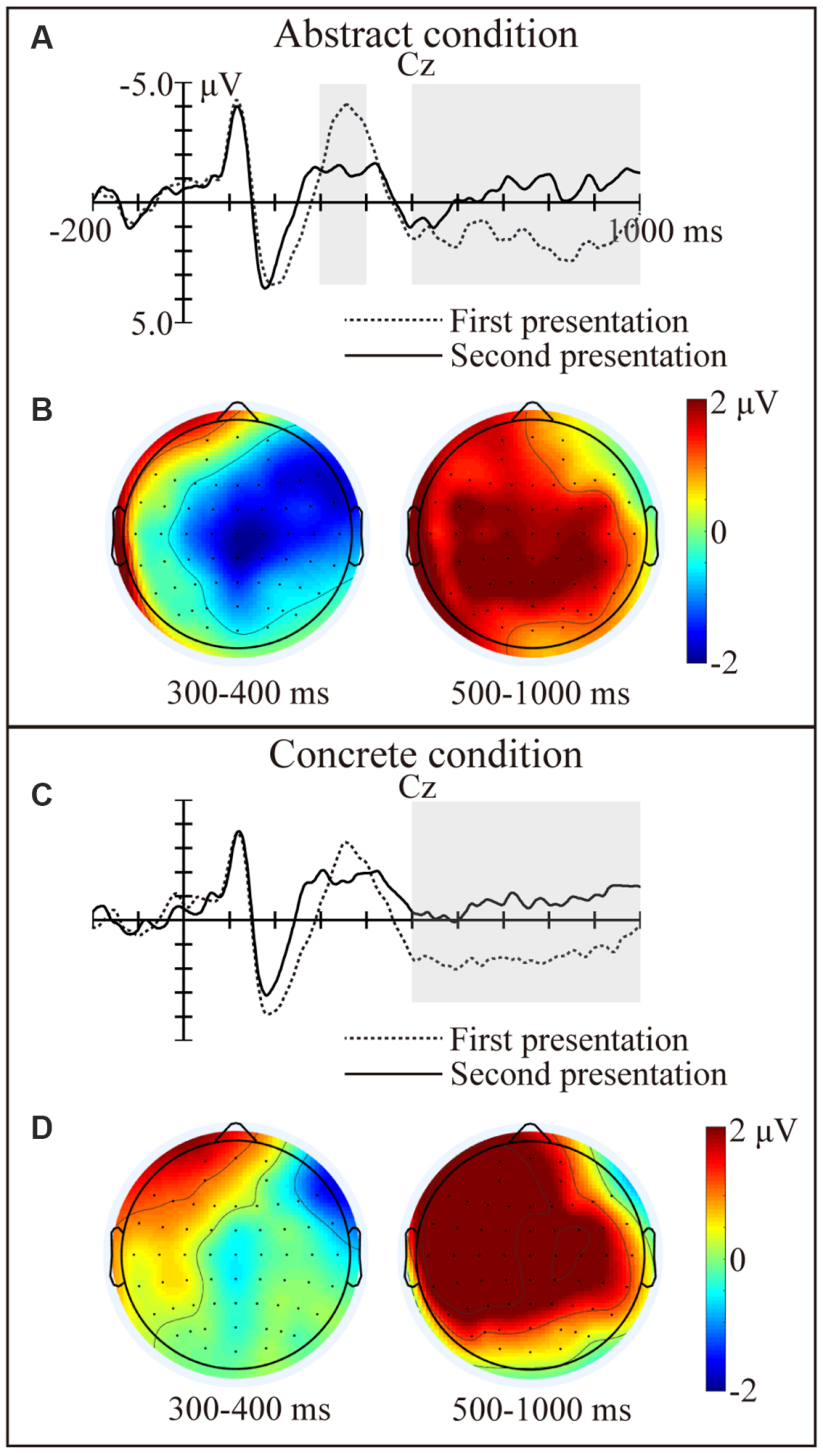
ERP results in the learning phase. Grand average waveforms elicited by the novel words in the learning phase in the abstract condition **(A)** and concrete condition **(C)** at Cz electrode. Topographies showing the N400 and LPC effects (first presentation vs. second presentation) in the abstract condition **(B)** and concrete condition **(D)**. The solid lines represent the novel words presented for the first time. The dotted lines represent the novel words presented for the second time.

The grand average waveforms elicited by the novel words in the learning phase at CZ electrode are presented in Figure 2. For the learning phase, in the time window of 300–400 ms, we found a significant interaction between concreteness and time [*F*_(1,23)_ = 11.23, *p* = 0.003, *η_p_^2^* = 0.328]. The following simple-effects tests showed that the abstract novel words elicited smaller N400s for the first time than the second time [*F*_(1,23)_ = 6.60, *p* = 0.017, *η_p_^2^* = 0.223]. This effect was absent for the concrete novel words [*F*_(1,23)_ = 0.01, *p* = 0.910, *η_p_^2^* = 0.001]. Meanwhile, the abstract novel words elicited larger N400s than the concrete novel words for the first time [*F*_(1,23)_ = 4.47, *p* = 0.045, *η_p_^2^* = 0.163], but not for the second time [*F*_(1,23)_ = 4.25, *p* = 0.051, *η_p_^2^* = 0.156]. In the time window of 500–1,000 ms, there was a significant main effect of time [*F*_(1,23)_ = 43.38, *p* < 0.001, *η_p_^2^* = 0.653] and a significant interaction between time and hemisphere [*F*_(2,46)_ = 5.72, *p* = 0.006, *η_p_^2^* = 0.199]. The following ANOVAs showed that the novels words elicited larger late positivities for the first time than the second time in the left [*F*_(1,23)_ = 83.33, *p* < 0.001, *η_p_^2^* = 0.784], middle [*F*_(1,23)_ = 29.30, *p* < 0.001, *η_p_^2^* = 0.560], and right [*F*_(1,23)_ = 16.60, *p* < 0.001, *η_p_^2^* = 0.419] regions.

**Figure 3:**
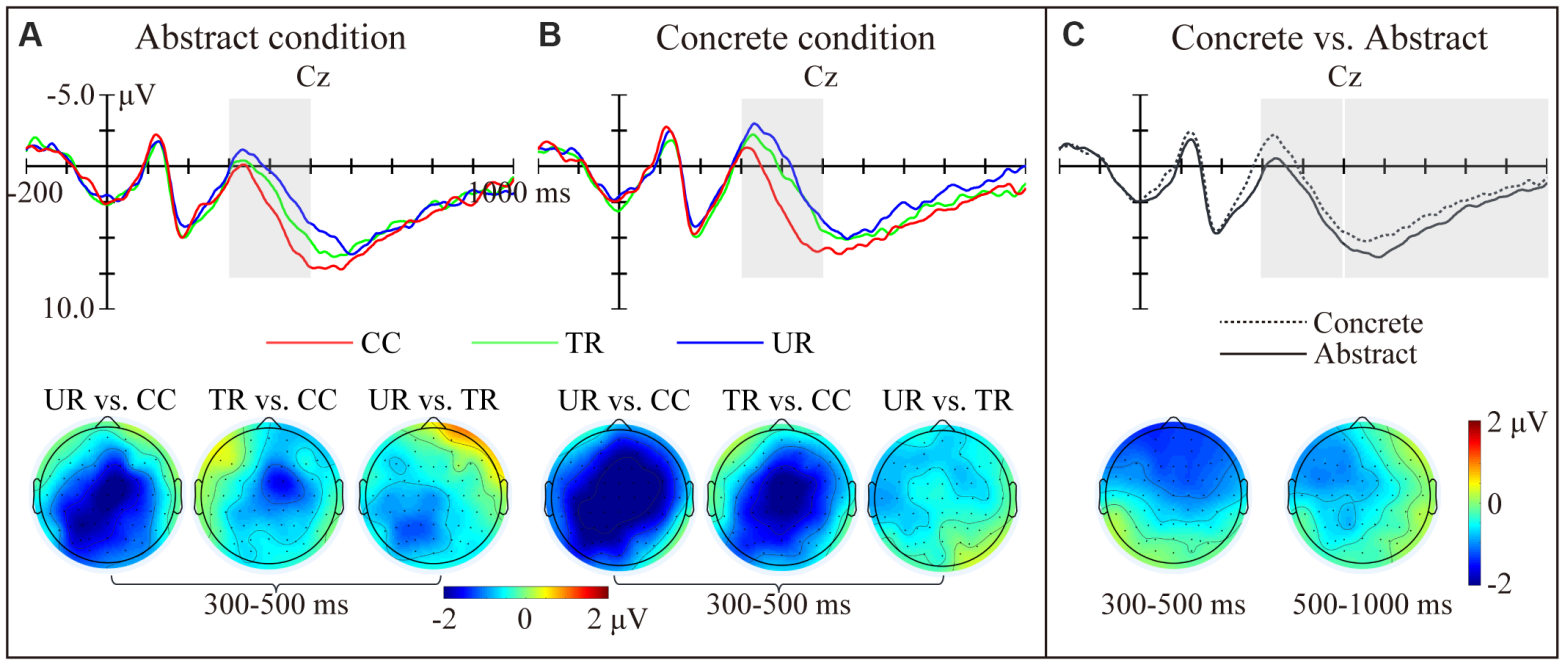
ERP results in the lexical decision task. Grand average waveforms elicited by the target words and topographies showing the N400 effects in the abstract condition **(A)** and concrete condition **(B)** at Cz electrode. Panel **(C)** presents the grand average waveforms elicited by the concrete and abstract targets at Cz electrode, as well as the topographies showing the N400 and LPC effects in response to the conceptual concreteness. The red lines represent the corresponding concepts (CC). The green lines represent the thematically related words (TR). The blue lines represent the unrelated words (UR). The solid line represents the concrete condition. The dotted line represents the abstract condition.

Figure 3 presents the grand average waveforms elicited by the target words at CZ electrode. For the testing phase, in the N400 time window, we found a significant main effect of target condition [*F*_(2,46)_ = 16.63, *p* < 0.001, *η_p_^2^* = 0.420]. Pair-wised comparisons revealed that the CC targets elicited the smallest N400s, the TR targets the moderate, and the UR targets the largest [CC vs. UR: *t*_(23)_ = 4.52, *p* < 0.001; TR vs. UR: *t*_(23)_ = 3.08, *p* = 0.016; CC vs. TR: *t*_(23)_ = 3.70, *p* = 0.004]. We also found a significant main effect of concreteness [*F*_(1,23)_ = 20.65, *p* < 0.001, *η_p_^2^* = 0.473], with the concrete condition eliciting larger N400s than the abstract condition. The interactions between concreteness and hemisphere [*F*(2,46) = 3.52, *p* = 0.038, *η_p_^2^* = 0.133], as well as between concreteness and anteriority [*F*_(2,46)_ = 16.14, *p* < 0.001, *η_p_^2^* = 0.412] were also significant. The following ANOVAs showed that the concrete targets elicited larger N400s than the abstract targets over the whole brain regions [Left: *F*_(1,23)_ = 10.73, *p* = 0.003, *η_p_^2^* = 0.318; Middle: *F*_(1,23)_ = 19.41, *p* < 0.001, *η_p_^2^* = 0.458; Right: *F*_(1,23)_ = 18.91, *p* < 0.001, *η_p_^2^* = 0.451; Anterior: *F*_(1,23)_ = 32.08, *p* < 0.001, *η_p_^2^* = 0.582; Central: *F*_(1,23)_ = 18.07, *p* < 0.001, *η_p_^2^* = 0.440] except the posterior region [*F*_(1,23)_ = 2.66, *p* = 0.117, *η_p_^2^* = 0.104]. In the time window of 500–1,000 ms, only a significant main effect of concreteness was found [*F*_(1,23)_ = 8.68, *p* = 0.007, *η_p_^2^* = 0.274], with the abstract targets eliciting larger late positivities than the concrete targets.

## 4. Discussion

The current study investigated the influence of conceptual concreteness on reading acquisition and integration of novel words into semantic memory. During contextual reading, the abstract novel words elicited a larger N400 than the concrete novel words for the first presentation. In the memory test, concrete novel words were better recollected than abstract novel words. These results indicated that the meaning of abstract novel words was derived more difficultly and more susceptible to forgetting than that of concrete ones. During the testing phase, both behavioral and ERP results revealed semantic integration of novel words into semantic network via thematic relations, as indicated by the graded RTs and accuracy as well as N400s for the CC, TR and UR targets, regardless of conceptual concreteness.

### 4.1. Modulation of concreteness on reading acquisition of novel words

In contextual reading, the abstract novel words elicited a larger N400 than the concrete novel words for the first presentation. N400 is a well-established component, indicating the easiness of semantic processing such as semantic retrieval or semantic integration (Lau et al., 2008; Kutas and Federmeier, 2011). Semantic violation would elicit a larger N400 during language comprehension (Kutas and Hillyard, 1980). When participants encountered the novel words in the first sentences, they would experience a semantic violation because the CC targets were strongly expected as indicated by their high cloze probabilities. Therefore, participants should retrieve semantic information (i.e., the CC targets) in semantic memory for the novel words. Due to the more consistency for words with more sensory-based representations and more variations for words with more language-based representations (Wang and Bi, 2021), the larger N400s for the abstract than concrete novel words indicated more difficulty in deriving the meaning of abstract novel words. This result was in line with the prediction of the dual coding theory that the concrete concepts would be better retrieved than abstract concepts with the support of both the verbal and imagery systems (Paivio, 1986, 1991).

However, this result seems to contradict the pretest results that showed no difference between the predictabilities of the corresponding concepts of concrete and abstract novel words (80.80 and 81.06% for concrete and abstract conditions, respectively). This contradiction between the ERPs and behavioral measures might be attributed to the different sets of cognitive operations which were caught by the two techniques. The ERPs reflect the online semantic processing of the stimuli, while the behavioral measures are associated with a series of cognitive processes, such as semantic access, decision making and motor operations within a relatively long-time duration (Holcomb, 1993; Chen et al., 2014). Therefore, without time pressure, the difficulty of deriving meaning of abstract novel words may not be reflected by the cloze probability test.

In addition, the novel words presented for the first time elicited larger late positivities than the second time in both conditions. This later effect, in terms of LPC or P600, in sentence comprehension is associated with semantic re-analysis of the perceptual input which is in conflict with the contextual prediction (Kuperberg, 2007; Brouwer et al., 2012; Kuperberg et al., 2020). As discussed above, the novel words in the first sentences were semantically anomalous compared with the corresponding concepts of novel words which were predicted based on the preceding contexts. After retrieving the meaning of novel words, participants needed to re-analyze the novel words by associating the corresponding concepts to them to make sense of the sentences. When the novel words were encountered at the second sentences, this semantic re-analysis was easier relative to that in the first sentences, resulting in smaller late positivities. In particular, the later effect was not influenced by conceptual concreteness. We postulated that the difficulty in semantic retrieval might have been resolved in the N400 time window. After the corresponding concepts were retrieved, the semantic re-analysis was not that demanding with no differences between the concrete and abstract contexts due to the strong constraints in both conditions. This later effect, to some extent, can be considered as a supportive evidence of context availability hypothesis in contextual reading at electrophysiological level. Combined with the N400 results, the ERPs in contextual reading indicate a cognitive neurodynamic processing of concrete and abstract words during sentence comprehension.

The influence of conceptual concreteness manifested in the lexical decision task. The concrete target words elicited larger N400s and smaller late positivities than the abstract ones, regardless of the semantic relatedness with the prime words. Previous studies have reported this larger long-lasting negativities for concrete words than for abstract words in a variety of processing tasks (Kounios and Holcomb, 1994; Holcomb et al., 1999; West and Holcomb, 2000; Kanske and Kotz, 2007; Huang et al., 2010; Adorni and Proverbio, 2012; Barber et al., 2013; Palmer et al., 2013; Bechtold et al., 2018, 2022). The long-lasting negativity effect includes an initial N400 component and a late N700 component. Holcomb et al. (1999) extended the dual coding theory and the context availability theory and proposed that concrete concepts are supported by superior linguistic information and an additional imagery-based system relative to abstract concepts. The N400 effect in response to conceptual concreteness is proposed to reflect stronger involvement of semantic activation or integration for concrete (vs. abstract) words which is in line with context availability theory; consistent with the dual coding theory, the N700 effect is associated with enhanced retrieval of visual imagery information (Kounios and Holcomb, 1994; Holcomb et al., 1999; Bechtold et al., 2018, 2022). It is worth noting that, in line with previous studies, the current study obtained a fronto-central N400 effect in response to conceptual concreteness in single-word processing (Barber et al., 2013; Palmer et al., 2013). While words were accompanied by contexts (Holcomb et al., 1999; West and Holcomb, 2000; Bechtold et al., 2022), the N400 effect distributed broadly or centro-parietally, which might be attributed to the high demands of integrating the words into contexts (Bechtold et al., 2022).

In addition, different from the ERP results, in the lexical decision task the behavioral results did not show the concreteness effect, that is, no difference in RTs or accuracies between the concrete and abstract conditions. These results are in line with the context availability hypothesis which predicts that the concreteness effect would disappear in the case words were embedded in contexts, especially in highly constraining contexts (Schwanenflugel et al., 1988; van Hell and de Groot, 2008). It was shown that highly constraining sentences in the current study facilitated the semantic access of both the concrete and abstract novel words, and in turn facilitated the implicit semantic processing of the target words. However, in the explicit semantic processing, namely cued-recall memory task, the performance for the concrete words was better than the abstract words. This may be ascribed to the strategies that participants used in the task. At the beginning of the experiment, they were told that there would be a memory test for the novel words. In this case they had to try to retain the corresponding concepts of novel words. As discussed above, in the support of (extended) verbal and imagery information for the concrete concepts, concrete novel words would be recollected better than abstract ones. It is due to the memory test, participants would be more sensitive to the conceptual concreteness, resulting in the N400 and N700 effects for the target words in the current study. Taken together, all these results suggest a modulation of conceptual concreteness on reading acquisition of novel word.

### 4.2. Similar semantic integration of abstract and concrete novel words

In the lexical decision task, the CC targets elicited the smallest N400s than other targets, and participants responded fastest to the CC targets with the highest accuracy. These results, along with the observed effects in the learning phase, indicated that the meaning of novel words was acquired in contextual reading, in line with previous studies exploring novel word learning in contextual reading (Mestres-Missé et al., 2007; Borovsky et al., 2012, 2013; Chen et al., 2014; Ding et al., 2017a,b; Zhang et al., 2017, 2019).

In addition, compared to the UR targets the TR target elicited smaller N400s with shorter RT and higher accuracy, indicating the establishment of thematic relations between novel and existing words in semantic memory. Using concrete words as stimuli, previous studies found that novel words could be integrated into semantic network through thematic relations (Zhang et al., 2017, 2019). In addition, this kind of semantic integration was modulated by the richness of learning context content. In contexts describing a single episode, novel words could only connect with words through thematic relation gained in the learning episodes; while in multiple episodic contexts, could connect with more words through extended thematic relations, even not implicated in the learning contexts (Zhang et al., 2019). In the current study, two sentences in a context depicted two episodes for the novel word, and the thematically related word in the testing phase was not related to the learning episodes. Therefore, the results of the present study did replicate the previous study in the concrete condition (i.e., Zhang et al., 2019).

Most importantly, it was found that the integration of novel words was not modulated by conceptual concreteness. In other words, both concrete and abstract novel words were integrated into semantic memory in a similar way through thematic relations. The findings are not predicted by different representational frameworks theory, which posits that abstract concepts connect with other concepts mainly via thematic relations while concrete concepts via taxonomic relations. According to Crutch and Warrington (2010), the theory was a graded but not binary model. Specifically, both concrete and abstract concepts connect with other concepts through similarity-based (taxonomic) and associative (thematic) information; the dependence on taxonomic or thematic relations varies along the continuum of concreteness, with more concrete concepts being more dependent on taxonomic representation and more abstract concepts more on thematic organization. In the current study, the corresponding concepts of concrete and abstract novel words were significantly different in conceptual concreteness on a 7-point Likert scale (6.15 and 2.73 for concrete and abstract conditions, respectively). The abstract novel words would connect with known words in semantic memory relying on much more thematic relations than the concrete novel words, which would be reflected by different semantic priming effects for the thematically related target words in abstract and concrete conditions. However, the observed smaller N400s for the thematically related (vs. unrelated) words did not interact with conceptual concreteness.

Previous studies have found that novel words learned in different types of contexts are integrated into semantic memory via different sematic relations (Ding et al., 2017a; Zhang et al., 2017, 2019). For instance, novel words are integrated into semantic memory either in contexts describing semantic features of concepts via taxonomic relations (Ding et al., 2017a) or in episodic contexts depicting the events or episodes via thematic relations (Zhang et al., 2017). One might argue that the similar integration through thematic relations for concrete and abstract novel words could be due to the learning episodic contexts, which emphasized the thematic associations among the concepts in the events. Nevertheless, a subsequent study has shown that novel words learned from episodic contexts could be also integrated into semantic memory via taxonomic relations (Zhang et al., 2019). Especially, our previous study using the same learning contexts with the current study found similar semantic integration of concrete and abstract novel words via taxonomic relations (Ding et al., 2017b). The results of our previous and current studies could not provide evidence for an asymmetric semantic integration of concrete and abstract concepts into semantic memory via taxonomic and thematic relations. Although the concrete novel words were learned better than the abstract ones, as predicted by the (extended) dual coding theory, the concrete and abstract novel words could establish both taxonomic and thematic relations with semantic memory via episodic contexts. Combined with the learning effects, it can be concluded that the conceptual concreteness only influences the learning processes of novel words but not the way the novel words are integrated into semantic memory.

However, the similar integration pattern of concrete and abstract novel words could not completely rule out the different dependence on taxonomic and thematic relations for concrete and abstract concepts due to the following reason. In the lexical decision task, only one word which was typically thematically related to the novel word was selected as stimuli. Due to the balance between semantic relatedness of the TR target words to both concrete and abstract novel words (CC target words in the pretest), the observed semantic priming effects were not significantly different in the two conditions. If using the blocked-translation paradigm in which a series of thematically related words served as competitors (e.g., Zhang et al., 2013), the semantic integration of novel words as indicated by semantic interference might be different between the concrete and abstract conditions.

### 4.3. Implications on L2 reading acquisition

The contextual learning paradigm used in the current and previous studies (e.g., Mestres-Missé et al., 2007, 2008; 2010; Borovsky et al., 2010; Chen et al., 2014; Ding et al., 2017a,b; Zhang et al., 2017, 2019; Liu et al., 2019) associated novel words with known concepts in semantic memory. This paradigm can be treated as a simulation of L2 vocabulary leaning, in which the concepts of novel words already exist in learner’s semantic memory (Mestres-Missé et al., 2007, 2014; Ferreira et al., 2015). Based on the findings of the current and our previous study (Ding et al., 2017b), the novel words could be learned rapidly and integrated into semantic memory via both taxonomic and thematic relations irrespective of conceptual concreteness. In addition, Zhang et al. (2019) found that in multiple episodic contexts novel words could be integrated into semantic memory via extended thematic relations and taxonomic relations. Taken together, all these results indicate that embedding words in episodic contexts, especially in multiple episodic contexts, will facilitate the learning efficiency for L2 vocabulary learning.

However, the generalization of the conclusion should be cautious. First, although previous studies revealed rapid acquisition of novel words in contextual learning paradigm in different languages, such as English (e.g., Borovsky et al., 2010, 2012, 2013), Spanish (e.g., Mestres-Missé et al., 2007, 2014), Chinese (e.g., Ding et al., 2017a,b; Zhang et al., 2017, 2019) and so on, only in Chinese the modulations of contextual information on the integration of novel words into semantic memory were examined here. Whether the facilitation effect of multiple episodic contexts on L2 vocabulary learning could be found in various languages needs to be further addressed. Second, individual differences such as age might modulate the observed effects. The weight of taxonomic and thematic relations in conceptual system organization changes during life span development. For instance, during children’s growing up, there is a developmental shift from thematic to taxonomic thinking; and thematic thinking gains more weight for aging people (for a review see Mirman et al., 2017). The participants recruited in the current study were adults which may rely on both taxonomic and thematic relations. Third, there has been evidence for the modulation of sentential constraint and L2 proficiency level on L2 word learning (Pulido, 2003; Borovsky et al., 2010; Elgort et al., 2015; Ma et al., 2015, 2016; Chen et al., 2017). Only in highly constraining sentences could the meaning of L2 novel words be derived (Ma et al., 2015), and higher-proficiency L2 learners outperformed than lower-proficiency L2 learners (Ma et al., 2015; Chen et al., 2017). Most of sentences used in these studies are highly constraining and presented in L1 for participants, who could be treated as high proficiency L2 language learners. Therefore, using weakly constraining sentences and recruiting L2 learners with different language proficiency levels and ages to investigate reading acquisition and semantic integration of novel words would increase the generalizability of the current conclusion in future studies. In addition, future studies are also needed due to the following limitation of the current study. In order to compare the current study with our previous study (i.e., Ding et al., 2017b), we used the same learning procedure in which the same novel word was repeated six times to each participant. Although the presenting order was counterbalanced across conditions, the repetition effects could not be completely excluded. Constructing more items and using a Latin square design will increase the robustness of the current conclusion.

## 5. Conclusion

The current study explored the modulation of conceptual concreteness on reading acquisition and semantic integration of novel words using contextual learning paradigm. During contextual reading, the meaning of abstract novel words was inferred with more difficulty than concrete novel words. In a cued-recall memory test assessing the learning effects of novel words, concrete novel words were preserved better than abstract ones. All these results, consistent with the (context-extended) dual coding theory, indicate the support of verbal and imagery information for concrete words processing. Meanwhile, both concrete and abstract novel words could construct thematic relations with known concepts in semantic memory, which is in conflict with the different representational frameworks theory under this circumstance (i.e., in lexical decision task). The results suggest a modulation of conceptual concreteness on reading acquisition but not on the type of semantic relations through which novel words are integrated into semantic memory.

## Data availability statement

The raw data supporting the conclusions of this article will be made available by the authors, without undue reservation.

## Ethics statement

The studies involving human participants were reviewed and approved by Ethics Committee of the Institute of Psychology, Chinese Academy of Sciences. The patients/participants provided their written informed consent to participate in this study.

## Author contributions

JD: formal analysis, data curation, writing–original draft, writing–review and editing, and funding acquisition. PL: methodology, software, investigation, and visualization. XG: investigation and writing–review and editing. YY: conceptualization, writing–review and editing, supervision, project administration, and funding acquisition. All authors contributed to the article and approved the submitted version.

## Funding

This work was supported by the National Natural Science Foundation of China (Grant Nos. 31900762 and 61433018).

## Conflict of interest

The authors declare that the research was conducted in the absence of any commercial or financial relationships that could be construed as a potential conflict of interest.

## Publisher’s note

All claims expressed in this article are solely those of the authors and do not necessarily represent those of their affiliated organizations, or those of the publisher, the editors and the reviewers. Any product that may be evaluated in this article, or claim that may be made by its manufacturer, is not guaranteed or endorsed by the publisher.
